# Microbial Consortia-Dependent Evolution of Physicochemical, Compositional and Functional Properties in Kombucha Fermentation

**DOI:** 10.3390/foods15142445

**Published:** 2026-07-09

**Authors:** Izaskun Martín-Cabrejas Pina, Sofía Montoro-Espada, Diego Morales

**Affiliations:** Departmental Section of Galenic Pharmacy and Food Technology, Veterinary Faculty, Complutense University of Madrid, Av. Puerta del Hierro, s/n, 28040 Madrid, Spain; sofmonto@ucm.es

**Keywords:** tea kombucha, SCOBY, phenolic compounds, antioxidant activity, kombucha microbiota

## Abstract

In recent decades, kombucha, a beverage produced through the fermentation of tea leaves by a symbiotic culture of bacteria and yeasts (SCOBY), has gained considerable popularity and attracted increasing scientific interest. However, the influence of SCOBY composition on kombucha characteristics remains insufficiently explored. In the present study, green tea kombuchas fermented with four different SCOBYs (SCs) were monitored over 21 days through physicochemical (pH), biochemical (total soluble solids (TSS), ethanol, proteins, and phenolic compounds), and microbiological (yeasts, acetic acid bacteria, and lactic acid bacteria) analyses. Based on these parameters, the most suitable bottling time was established for each kombucha (7 days for SC3 and SC4; 10 days for SC1 and SC2), considering acceptable and safe pH values, reduced TSS levels (4.3–4.6 °Brix), ethanol concentrations below 1.2%, and increased protein and phenolic compound contents, all of them differently affected by the SC used. At the selected bottling stages, the kombuchas exhibited DPPH^●^ and ABTS^●+^ radical scavenging capacities (TEAC values up to 7.7 and 36.7 µmol/mL, respectively) and inhibitory activity against an *Escherichia coli* strain. These preliminary findings suggest the impact of SC composition and support further studies involving advanced phenolic, microbiological, and functional characterization approaches.

## 1. Introduction

Kombucha is a brewed beverage traditionally produced from a sweetened infusion of tea (*Camellia sinensis*) leaves fermented by a symbiotic culture of bacteria and yeasts (SCOBY). In recent years, it has gained popularity as a perceived healthier alternative to soft drinks and sugar-sweetened and/or alcoholic beverages. Although its expansion in Western countries is relatively recent, its origin is commonly traced back to around 220 BC in the Manchuria region of northeastern China. However, it is in the last few decades that kombucha consumption has experienced remarkable global growth, accompanied by increasing scientific interest, as reflected in the rising number of studies investigating the characteristics and properties of this fermented product [1,2,3].

One of the major current challenges is to achieve a deeper understanding of the relationship between kombucha characteristics and its microbiota, which is distributed between the liquid phase and the ecosystem formed within the SCOBY (SC) itself, primarily composed of cellulose and diverse microbial species. This symbiotic culture responsible for fermentation may include, depending on the fermentation stage, yeasts (*Saccharomyces* spp., *Schizosaccharomyces* spp., *Pichia* spp., etc.), acetic acid bacteria (AAB; *Acetobacter* spp., *Gluconobacter* spp., *Komagataeibacter* spp., etc.), and lactic acid bacteria (LAB; *Lactobacillus* spp., *Lactococcus* spp., *Oenococcus* spp., etc.) [4,5,6]. This microbial community is complex and varies according to the raw material used (tea leaves or alternative substrates), the sugar source, and fermentation conditions (temperature, time, starter culture, pH, etc.), indicating that no unique or standardized microbiota can be defined for kombucha [7,8].

Furthermore, the relationship between kombucha properties and its microbiota is bidirectional. While the physicochemical conditions at each fermentation stage may promote or inhibit the growth of specific microorganisms, the presence, abundance, and metabolic activity of the microbial populations also modulate the physicochemical, biochemical, and functional characteristics of the beverage [9,10].

Regarding the functional properties of tea kombucha, numerous biological activities have been attributed to this beverage, including antioxidant, antimicrobial, anti-inflammatory, antitumoral, hypolipidemic, and microbiota-modulating effects. Nevertheless, most of these findings derive from in vitro studies, with relatively few validations conducted in animal models or clinical trials [1,11].

Accordingly, the aim of the present study was to produce green tea kombucha using four different SCs and to monitor, over 21 days of fermentation, their physicochemical (pH) and biochemical parameters (soluble sugars, ethanol, soluble proteins, and total phenolic compounds), as well as the evolution in composition and population levels of the main microbial groups (yeasts, AAB, and LAB). In addition, the in vitro antioxidant and antimicrobial activities of the beverages exhibiting the most favourable characteristics were evaluated.

## 2. Materials and Methods

### 2.1. Biological Material

Commercial green tea (Auchan, Villeneuve d’Ascq, France) was purchased from a local supermarket. Tea bags containing buds and leaves of *Camellia sinensis* were used for infusion preparation. Four distinct SCs were used: SC1, prepared in the laboratory from non-pasteurized commercial kombucha following the protocol described by Morales et al. (2023) [7]; SC2, obtained from “Oh My Kefir!”; and SC3 and SC4, both provided by small-scale kombucha producers.

For the antimicrobial activity studies, *Escherichia coli* (DH5α strain) (Thermo Fisher Scientific, Waltham, MA, USA), *Staphylococcus aureus* (CECT 86–NCTC 8532 strain), and *Listeria innocua* (CECT 8848–SA1 strain) from the Spanish Type Culture Collection (CECT) were employed.

### 2.2. Reagents

Solvents such as methanol and absolute ethanol were acquired from Panreac (Barcelona, Spain), as well as sodium carbonate (Na_2_CO_3_). Hydrochloric acid (HCl, 37%), Folin–Ciocalteu’s phenol reagent, Bradford reagent, 2,2′-azino-bis(3-ethlybenzothiazoline-6-sulphonic acid (ABTS), potassium persulfate, gallic acid, 2,2-diphenyl-1-picrylhydrazyl (DPPH), bovine serum albumin, and Trolox were purchased from Sigma-Aldrich Quimica (Madrid, Spain). Brain Heart Infusion (BHI) broth, bacteriological agar, plate count agar (PCA), potato dextrose agar (PDA), Man, Rogose and Sharpe (MRS), and Glucose Yeast-Extract Calcium Carbonate (GYC) culture media were obtained from Condalab (Torrejón de Ardoz, Spain).

### 2.3. Kombucha Elaboration

The kombucha beverages were prepared in duplicate following the protocol of Morales et al. (2023), with minor modifications [7]. Briefly, green tea powder (12 g/L) was added to hot tap water (80 °C) along with white cane sugar (70 g/L). The mixture was vigorously stirred to obtain a homogeneous suspension and then cooled to room temperature (24.0 ± 0.5 °C) before inoculation with SC1, SC2, SC3, or SC4 (40 g/L) and 10% (*v*/*v*) of the medium in which the SC had been previously maintained (“old kombucha”), ensuring an initial pH (<4.2) that prevented the growth of undesirable microorganisms. The kombucha recipients were kept open, covered with filter paper, and stored in the dark at room temperature (24.0 ± 0.5 °C) for 21 days.

### 2.4. Physicochemical Characterization of Kombuchas: pH

The pH levels were determined using a Hanna pH metre (model HI 4211; Hanna Instruments, Eibar, Spain) on days 0, 3, 7, 10, 14, and 21.

### 2.5. Biochemical Characterization of Kombuchas

Aliquots of each kombucha were collected on days 0, 3, 7, 10, 14, and 21 of fermentation and stored at −20 °C until their subsequent use in the various analyses and biochemical composition determinations.

#### 2.5.1. Total Soluble Solids (TSS) Content

Total soluble solids (TSS) content was measured in the kombuchas using a handheld ATC refractometer (Brouweland, Beverlo, Belgium), and the results were expressed in °Brix.

#### 2.5.2. Ethanol Content

Ethanol content in the kombuchas was quantified using a handheld ALL001 refractometer (Allmeter, London, UK), and the results were expressed as alcohol by volume (% *v*/*v*).

#### 2.5.3. Soluble Proteins Content

The total soluble protein concentration was determined using the Bradford method reagents (Sigma-Aldrich, Madrid, Spain) according to the instruction manual, using bovine serum albumin (0.0125 to 0.5 mg/mL) as the standard for quantification.

#### 2.5.4. Total Phenolic Content

Total phenolic compounds were quantified using the Folin–Ciocalteu method, adapted for kombucha samples by Morales et al. (2023), with slight modifications [7]. Each kombucha sample (50 µL) was mixed with 1.48% HCl (133 µL) and methanol (67 µL), and the mixture was centrifuged at 10,000 rpm for 2 min. The supernatants were then combined with 1 mL of 2% (*w*/*v*) Na_2_CO_3_ and, after a 3 min incubation, 25 µL of Folin–Ciocalteu reagent was added. Finally, the mixture was incubated in the dark for 30 min, and absorbance was measured at 750 nm. Gallic acid (0.0156–0.75 mg/mL) was used as the standard for quantification.

### 2.6. Microbiological Analyses

To quantify the microbial populations present in the different kombucha samples at various fermentation times (0, 3, 7, 10, 14, and 21 days), 4 culture media were used: PCA for the enumeration of aerobic mesophilic microorganisms (AMM); MRS agar for lactic acid bacteria (LAB); GYC agar for acetic acid bacteria (AAB); and PDA for yeasts. All media were prepared according to the manufacturer’s instructions. Kombucha samples collected on the corresponding fermentation day were appropriately diluted in sterile physiological saline, and 20 µL aliquots were surface-plated onto the different culture media. The inoculated plates were then incubated at 30.0 ± 1.0 °C for 24–72 h.

### 2.7. In Vitro Biological Activities

To perform the bioactivity analyses, the most appropriate fermentation time point was selected for each SCOBY based on kombucha pH, as well as its biochemical and microbiological composition. Accordingly, day 10 was chosen for SC1 and SC2, and day 7 for SC3 and SC4.

#### 2.7.1. DPPH^●^ and ABTS^●+^ Scavenging Activities

The obtained kombucha samples were diluted (1.56–50 µL/mL) and evaluated for their DPPH^●^ and ABTS^●+^ scavenging activities. The dilutions were combined with a methanolic DPPH^●^ solution (76 mM) or an aqueous ABTS^●+^ solution (7 mM; the ABTS radical was chemically generated using 2.47 mM potassium persulfate), following the protocols of Mau et al. (2001) and Re et al. (1999), respectively, as adapted by Morales et al. (2018) [12,13,14]. For the DPPH^●^ assay, absorbance at 517 nm was measured after 30 min incubation at room temperature in the dark. In the case of the ABTS^●+^ assay, absorbance was read at 734 nm after 15 min of incubation under the same conditions. For both radicals, the half maximal inhibitory concentration (IC_50_) values were determined using the linear correlation obtained with increasing sample concentrations, and results were compared with Trolox to express antioxidant capacity as TEAC (Trolox equivalent antioxidant capacity) values.

#### 2.7.2. In Vitro Antimicrobial Activity

The antimicrobial activity of the selected kombucha samples was assessed using the disc diffusion assay. Each microorganism (*E. coli*, *S. aureus*, and *L. innocua*; 10^5^ ufc/mL) was suspended in semi-solid BHI medium containing bacteriological agar (0.7% *w*/*v*) and then poured into sterile Petri dishes. Sterile Oxoid^TM^ test discs (Thermo Fisher Scientific, Madrid, Spain) were placed onto the surface of the inoculated agar, and 20 µL of each extract was applied to the discs. Gentamicin (10 µg) was used as positive antimicrobial control (Oxoid^TM^ gentamicin test discs, Thermo Fischer Scientific, Madrid, Spain). The plates were subsequently incubated at 37 °C for 48 h. All samples were analyzed in triplicate, and antimicrobial activity was determined by measuring the diameter (mm) of the inhibition zones.

### 2.8. Statistical Analysis

Differences were evaluated at a 95% confidence level (*p* ≤ 0.05) using a one-way analysis of variance (ANOVA) followed by the Tukey multiple comparison test. Statistical analysis was performed using GraphPad Prism version 9.5.1 (GraphPad Software, San Diego, CA, USA).

## 3. Results and Discussion

### 3.1. Evolution of pH of Kombucha Beverages During Fermentation

**Figure 1:**
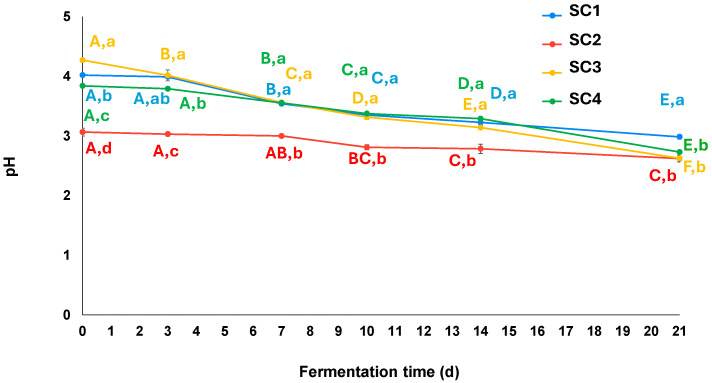
Evolution of pH values in kombuchas during fermentation (21 days) with SC1 (blue), SC2 (red), SC3 (orange), and SC4 (green). Different letters denote significant differences for the same SC at different fermentation times (A–F) and for different SCs at the same fermentation time (a–d) (one-way ANOVA, Tukey’s test, *p* ≤ 0.05).

A progressive decrease in pH was observed throughout fermentation in all kombucha samples, regardless of the SC used (Figure 1). This acidification was expected, as it results from the production of organic acids by SC-associated microorganisms, mainly acetic acid released by AAB through the oxidation of ethanol produced by yeasts during fermentation [15]. However, some differences were noted depending on the SCOBY employed. SC1, SC3 and SC4 exhibited similar trends, with initial pH values ranging from 3.84 to 4.27 and a pronounced decline over time, particularly in SC3-kombucha, which reached values as low as 2.63 after 21 days. In contrast, kombucha fermented with SC2 started at a significantly lower pH (3.07) and showed a more gradual decrease, reaching 2.62 after three weeks. This variation in acidification kinetics may be attributed to differences in the presence and abundance of AAB population and particular species [16]. Indeed, kombucha produced with SC2 showed the highest initial AAB levels (5.61 log CFU/mL). Moreover, the initial acidity of the “old kombucha” (i.e., the liquid medium in which the SCOBY is maintained prior to use) likely played a relevant role in these initial differences [1]. An important aspect of the obtained kombuchas is that their pH values remained, in most cases, within the range considered safe for human consumption (2.5–4.2). Values below 2.5 indicate excessively high acetic acid concentrations, whereas values above 4.2 may compromise microbiological safety due to an increased risk of undesirable microbial growth [7,16]. As observed, pH values did not fall below 2.5 during the 21-day fermentation period, and only the initial pH of kombucha fermented with SC3 slightly exceeded the upper safety threshold (4.27). Nevertheless, no growth of mould-like microorganisms (the main biological contaminants in these beverages) was detected in any sample [17].

In comparison with previously published data, similar pH reductions have been reported in green tea kombucha prepared with a microbial consortia (Supplementary Figure S1) comparable to SC3, decreasing from an initial pH of 3.7 to 2.6 after 21 days of fermentation. In that study, however, the decline was particularly abrupt between days 7 and 10, remaining relatively stable during the final two weeks [7]. Another study on green tea kombucha fermented with SCs dominated by the genus *Komagataeibacter* (AAB) reported pH decreases from 3.1 to 2.6 after 14 days [17]. Overall, the pH of these beverages typically falls within the aforementioned range, with variations influenced by both the SC composition and the raw materials used, which also contribute to the release of different organic acids during fermentation [1,18].

### 3.2. Evolution of Biochemical Composition of Kombucha Beverages During Fermentation

#### 3.2.1. Total Soluble Solids Content

Total soluble solids (TSS) content was estimated by measuring °Brix, as this parameter is widely used as a rapid proxy for total soluble sugars in fermented beverages. However, in kombucha, °Brix values should be interpreted as an approximation rather than a direct measurement of sugar concentration, because refractometric readings are influenced not only by sugars but also by other dissolved compounds generated during fermentation, including ethanol, acetic acid, and other organic acids. Nevertheless, °Brix measurements remain useful for comparative and process-monitoring purposes because sugars are still the predominant soluble solids in the matrix, and refractometry provides a rapid, reproducible, and non-destructive estimate of overall soluble solids’ evolution throughout fermentation. Previous studies on kombucha and other fermented beverages have similarly employed refractometric measurements as an indirect indicator of residual sugars and fermentation progress, while acknowledging the influence of ethanol and organic acids on refractive index values [19,20].

**Figure 2:**
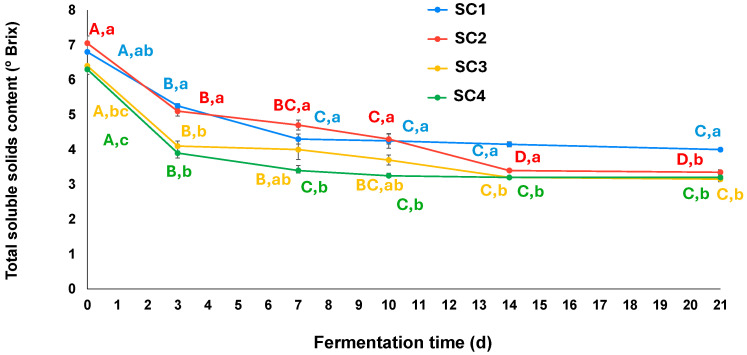
Evolution of total soluble solids content (°Brix) in kombuchas during fermentation (21 days) with SC1 (blue), SC2 (red), SC3 (orange), and SC4 (green). Different letters denote significant differences for the same SC at different fermentation times (A–D) and for different SCs at the same fermentation time (a–c) (one-way ANOVA, Tukey’s test, *p* ≤ 0.05).

At the beginning of the fermentation, the TSS content of the kombuchas analyzed ranged from 6.3 to 7.1 °Brix (Figure 2). Since all formulations contained the same amount of added sucrose (70 g/L) and green tea (12 g/L), the slight differences observed are likely attributable to the soluble solids contributed by the SC itself and, particularly, by the ‘old kombucha’. Accordingly, SC1 and SC2 produced kombuchas with higher initial °Brix values. Similar differences have previously been reported in studies using comparable SCs, where variations in the soluble carbohydrate content of kombuchas were already detected at the initial stage of fermentation [21,22].

Once the SC initiates its fermentative activity, a progressive decrease in TSS is observed in all kombuchas. This reduction is significantly more pronounced during the first 3 days of fermentation, coinciding with the period of highest yeast activity, during which sucrose is hydrolyzed and the resulting monosaccharides are metabolized as substrates for alcoholic fermentation. By day 3, TSS values had already decreased to between 3.9 and 5.3 °Brix. Thereafter, TSS content continued to decline at a slower rate, gradually stabilizing during the final stage of fermentation and reaching values between 3.2 and 4.0 °Brix after 21 days, with the kombucha produced using SC1 showing the highest TSS content. These residual TSS levels may be explained by the presence of sugars that cannot be metabolized by the SC microbial consortium, as well as of other dissolved solids, particularly organic acids such as acetic acid, which may also influence refractometric measurements [23].

These decreases in the TSS values of kombucha beverages have already been reported in previous studies. For instance, Wang et al. (2023) observed a progressive decline in TSS content in black tea kombucha, from 4.9 to 3.1 °Brix over 14 days of fermentation [19]. The hypothesis that these reductions are primarily attributable to the consumption of sucrose and its resulting monosaccharides by yeasts is further supported by other published findings. For example, Cardoso et al. (2020) reported that the initial sucrose content decreased from 5% to 3.5% and 2% in black tea and green tea kombuchas, respectively, after 10 days of fermentation [16]. Furthermore, in a previous study on green tea kombucha fermented with a microbial consortium similar to SC3, as used in the present work, a pronounced reduction in total carbohydrate content was observed, decreasing from 10.9% to 3.4% over 21 days of fermentation [7].

From a nutritional and health perspective, the TSS values recorded in the present study for kombucha beverages during the typical bottling period for conventional tea kombucha (7–10 days) ranged between 3.2 and 4.6 °Brix. In all cases, these values suggest substantially lower concentrations of soluble sugars than those commonly found in other beverages, such as fruit juices and carbonated soft drinks, which typically contain approximately 10–15% and 6–15% sugar, respectively [24,25]. Therefore, the green tea kombuchas produced in this study, particularly those fermented with SC3 and SC4, may represent a low-sugar alternative to other widely consumed beverages.

#### 3.2.2. Ethanol Content

Monitoring of the alcohol content of the kombucha beverages revealed that no detectable ethanol levels were present prior to the onset of fermentative activity by the SCs (Table 1).

However, alcoholic fermentation carried out by yeasts led to ethanol production, resulting in a progressive increase in its concentration during the first days of fermentation and reaching maximum levels between days 3 and 7. After peaking approximately one week into the fermentation process, ethanol concentrations began to decline to undetectable levels due to its oxidation by AAB, which produce acetic acid [26]. Indeed, a correlation between ethanol production and the final acidity of the kombucha beverages could be observed (R^2^ = 0.69 for maximum ethanol concentration vs. final pH), as SC2 and SC3 exhibited the highest ethanol concentrations during the intermediate stages of fermentation and also showed the lowest pH values at the end of the process (Table 1, Figure 1).

Considering that beverages containing more than 1.2% (*v*/*v*) alcohol must be labelled as alcoholic beverages and would therefore be excluded from any health claim declarations under European regulations [27], it is important to note that none of the kombuchas exceeded this threshold during the typical bottling period (days 7–10), with the exception of the kombucha fermented with SC2, which reached exactly 1.2% alcohol on day 7. In any case, if the aim is to position the developed kombuchas as nutritionally healthy beverages with potential beneficial properties, it is essential to monitor ethanol production and its subsequent removal in order to obtain a product that is, ideally, ethanol-free. To achieve this, it is important to select SCs containing yeasts that do not produce pronounced ethanol peaks, or AAB strains capable of efficiently oxidizing the ethanol produced without generating excessively acidic beverages. Furthermore, the bottling time could be reconsidered to ensure that the final product remains non-alcoholic.

The results obtained are consistent with those previously reported in the literature. This is the case for the black tea kombuchas produced by Ahmed et al. (2020), in which an increase in ethanol concentration was observed after 4 days of fermentation, reaching approximately 1.2%, followed by a subsequent decline due to the activity of AAB [26]. A similar pattern of ethanol increase and subsequent decrease was also reported in green tea kombuchas fermented with a microbial consortium comparable to SC3 used in the present study. However, significantly higher ethanol peaks were reached in that case, with concentrations up to 2.6% in day 4, before declining to undetectable levels after three weeks of fermentation [7].

#### 3.2.3. Soluble Proteins Content

**Figure 3:**
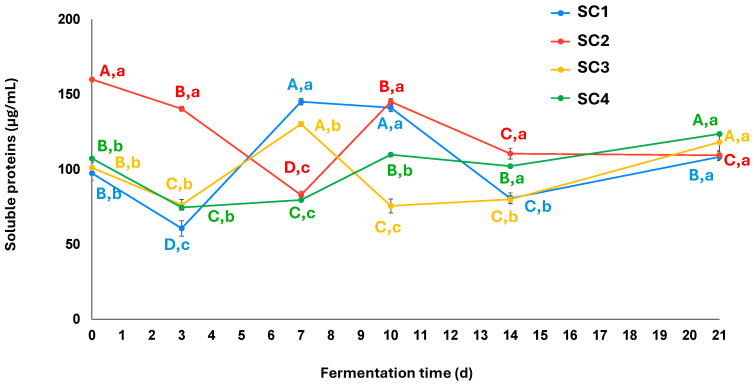
Evolution of soluble proteins content (µg/mL) in kombuchas during fermentation (21 days) with SC1 (blue), SC2 (red), SC3 (orange), and SC4 (green). Different letters denote significant differences for the same SC at different fermentation times (A–D) and for different SCs at the same fermentation time (a–c) (one-way ANOVA, Tukey’s test, *p* ≤ 0.05).

Traditional kombucha is not considered a particularly protein-rich beverage, as commercial tea kombuchas generally contain approximately 3 µg/mL of protein [28]. Nevertheless, the presence of peptides with biological activities at sufficiently high concentrations at the time of bottling may be of considerable interest [29]. Although the identification of such peptides and the validation of their bioactivity were beyond the scope of the present study, the soluble protein content of the kombucha beverages was monitored in order to determine the stages of fermentation at which protein levels were significantly higher. Prior to the onset of fermentation, the kombucha fermented with SC2 exhibited the highest soluble protein content (159.9 µg/mL), whereas the remaining kombuchas showed values ranging from 97.3 to 107.1 µg/mL (Figure 3).

These initial differences are likely attributable to proteins introduced by the SC itself and by the added old kombucha. During the first days of fermentation, a significant decrease in soluble protein content was observed, probably due to the starting low pH of the medium, which may promote protein degradation and precipitation in kombucha beverages [22]. This initial decline was followed by a recovery phase, reaching maximum values on day 7 for the SC3-kombucha (130.1 µg/mL) and on day 10 for the SC1- and SC-4 kombuchas (141.0 and 109.2 µg/mL, respectively), while the SC2-kombucha recovered to protein levels that were not significantly different from those measured at the beginning of fermentation. These increments might be linked to the release and solubilization of tea proteins due to microbial action and the low pH environment but also to the growth and activation of the SC microorganisms and the stimulation of their metabolism. These processes can contribute microbial proteins and peptides, which can also be released into the beverage and solubilized over time, leading to their accumulation as fermentation progresses [28,30]. Following this increase, a significant reduction was again observed, likely associated with the low pH and the consequent processes of protein degradation and precipitation [22].

Previous studies have also reported similar trends. Ahmed et al. (2020) recorded the maximum protein content in black tea kombucha on day 8 of fermentation, reaching 3.2 mg/mL, after which protein levels began to decline [26]. This phenomenon had already been described by Jayabalan et al. (2007), who reported an increase in protein concentration in green and black tea kombuchas from 100 µg/mL to 3000 µg/mL after 12 days of fermentation, followed by a subsequent decrease until day 18, when the fermentation process was terminated [31]. In both studies, the maximum protein concentrations were substantially higher than those observed in the present work, which were nevertheless markedly greater than the values previously reported for commercial tea kombuchas [28]. In this regard, it may be inferred that differences in protein content are primarily dependent on the SC employed and the fermentation conditions applied, as well as, to a lesser extent, on the characteristics of the tea used, which may also contribute soluble proteins and peptides to the final beverage [32].

#### 3.2.4. Total Phenolic Content (TPC)

**Figure 4:**
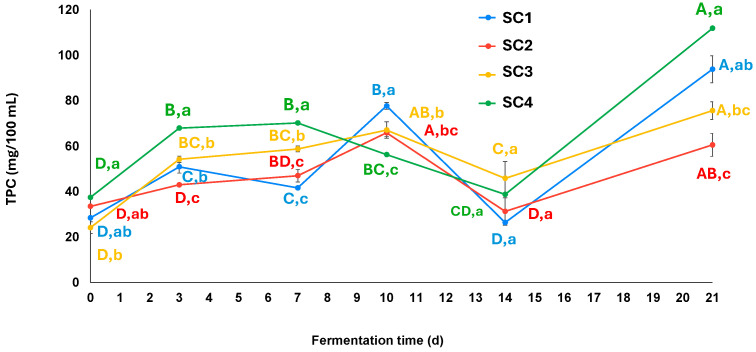
Evolution of total phenolic content (TPC, mg/100 mL) in kombuchas during fermentation (21 days) with SC1 (blue), SC2 (red), SC3 (orange), and SC4 (green). Different letters denote significant differences for the same SC at different fermentation times (A–D) and for different SCs at the same fermentation time (a–c) (one-way ANOVA, Tukey’s test, *p* ≤ 0.05).

As previously mentioned, the (poly)phenols present in tea kombucha are compounds of considerable interest due to their potential biological activities [4]. In the kombuchas developed in the present study, the initial TPC values were similar, ranging from 28.4 to 37.4 mg/100 mL (Figure 4), suggesting that these compounds were primarily derived from the raw material, which in all cases was green tea. Subsequently, an increasing trend was observed during the first days of fermentation, reaching an initial maximum coinciding with the typical bottling period. Thus, SC4-kombucha reached its first maximum TPC on day 7 (70.1 mg/100 mL), whereas SC1-, SC2-, and SC-3 kombuchas reached their highest values on day 10 (77.7, 65.9, and 67.0 mg/100 mL, respectively), although in the case of SC3-kombucha no significant differences were observed between days 7 and 10. These values were decisive in selecting the samples subjected to in vitro biological activity analyses. After day 10, a decrease in TPC was noticed, followed by a subsequent increase leading to significantly higher values after 21 days of fermentation, particularly in SC-1 and SC4-kombuchas, which reached 93.8 and 111.8 mg/100 mL, respectively.

The increases observed in total (poly)phenols concentrations are mainly attributed to the combined effects of decreasing pH and microbial activity, which promote the release of phenolic compounds from tea matrices and facilitate enzymatic reactions such as hydrolysis and depolymerization, thereby resulting in higher quantification by spectrophotometric methods. Conversely, TPC values may also be negatively affected by acid-induced degradation, as well as by aggregation, precipitation, or polymerization phenomena [7,33]. These fluctuations, characterized by alternating increases and decreases in the phenolic content of tea kombuchas, have previously been reported in fermentations using green tea and SCs similar to those employed in the present study [7], as well as in kombuchas prepared with SCs of different compositions using green and oolong tea [34].

### 3.3. Evolution of Microbial Composition of Kombucha Beverages During Fermentation

**Figure 5:**
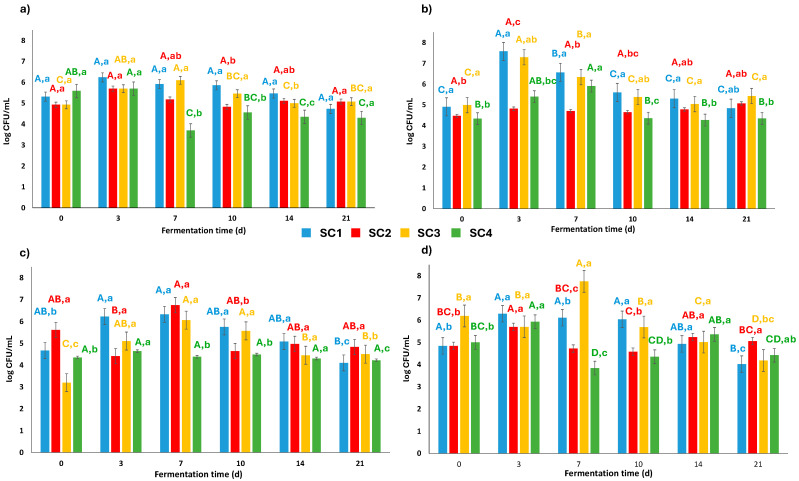
Evolution of microbial populations counts (log CFU/mL) in kombuchas during fermentation (21 days) with SC1 (blue), SC2 (red), SC3 (orange), and SC4 (green): (**a**) Aerobic mesophilic microorganisms; (**b**) yeasts; (**c**) acetic acid bacteria; (**d**) lactic acid bacteria. Different letters denote significant differences for the same SC at different fermentation times (A–D) and for different SCs at the same fermentation time (a–c) (one-way ANOVA, Tukey’s test, *p* ≤ 0.05).

It should be noted that the microbiological analyses performed in the present study were intended as a preliminary approach to monitor the general dynamics of the main microbial groups involved in kombucha fermentation. To this end, culture-dependent techniques employing non-selective or broadly selective media were used to estimate total aerobic mesophilic microorganisms (PCA), LAB (MRS), yeasts (PDA), and AAB (GYC) throughout the fermentation process. Although these methods provide valuable information regarding the overall evolution of microbial populations, they do not allow precise taxonomic identification or an exhaustive characterization of the microbial consortia present. Therefore, the results obtained (Figure 5) should be interpreted as an initial characterization that establishes the basis for future studies involving more specific and advanced identification approaches, such as molecular and metagenomic analyses, aimed at accurately defining the microbial composition and its functional implications during kombucha fermentation [28].

Beginning with the total mesophilic microorganism counts (Figure 5a), these results likely provide the least specific information regarding the microbial dynamics of kombucha fermentation, given that PCA is a highly general culture medium capable of supporting the growth of a broad range of microorganisms. Consequently, the counts obtained reflect the overall viable microbial population rather than the evolution of particular groups involved in the fermentation process. In contrast, the use of more selective media such as MRS, PDA, and GYC enables a more targeted monitoring of LAB, yeasts, and AAB, respectively. Moreover, some microbial groups commonly present in kombucha, including yeasts and LAB, may exhibit enhanced growth under the specific nutritional and environmental conditions provided by these selective media, thereby allowing a more representative estimation of their populations throughout fermentation [21]. With regard to their evolution throughout fermentation, all kombuchas exhibited similar initial concentrations, of approximately 5 log CFU/mL, remaining relatively stable during the first week of the process, with the exception of SC4-kombucha, which underwent a reduction to 3.7 log CFU/mL and maintained these levels until the end of fermentation. During the final two weeks of fermentation, significant reductions were also observed in SC3-kombucha, possibly as a consequence of reduced sugar availability more than because of the high acidity, since SC2-kombucha showed lower pH values. Indeed, SC3- and SC4-kombuchas exhibited the lowest TSS values from the first week of fermentation onwards (Figure 2).

Focusing now on yeasts (Figure 5b), and considering the limitation that other fungal microorganisms may also grow on the PDA medium employed, an increasing trend in microbial counts can be observed during the first days of fermentation. This pattern coincides with the period of maximum yeast activity, during which sucrose is utilized and metabolized through hydrolysis for alcoholic fermentation processes [35]. Particularly noteworthy was the marked increase in counts observed on day 3 for kombucha fermented with SC1 and SC3, which reached 7.6 and 7.3 log CFU/mL, respectively. It should also be highlighted that SC2-kombucha exhibited relatively constant counts of these microorganisms throughout the entire fermentation process, despite evidence of pronounced yeast metabolic activity reflected by its rapid and substantial ethanol production (Table 1). It is possible that this marked ethanol generation may itself exert an inhibitory effect on yeast growth and proliferation [36].

With regard to AAB, the highest counts were observed between days 3 and 7 of fermentation (Figure 5c), coinciding with the period of greatest ethanol availability. The highest population levels were detected in SC2-kombucha on day 7, reaching up to 6.8 log CFU/mL, which is consistent with the fact that this kombucha also exhibited the highest ethanol concentrations among all the beverages developed in the present study. As fermentation progressed, particularly from day 14 onwards, significant reductions in AAB populations were observed, presumably due to the depletion of ethanol in the medium. It is worth noting that this trend was not observed in SC4-kombucha, which maintained concentrations close to 4 log CFU/mL throughout the entire process and did not exhibit any significant population increase at any stage of fermentation. Referring again to Table 1, it can be inferred that the low ethanol production in this kombucha also limited the growth of AAB, which oxidize this molecule to produce acetic acid [35].

Finally, the concentrations of LAB in the kombuchas were also analyzed. Although they are not usually the microorganisms that receive the most attention in kombucha research, they can play a relevant role depending on the SC used and the fermentation conditions. They complement the activity of yeasts and AAB and contribute mainly to acidification, the aromatic profile, and potentially to the functional properties of the final beverages [36]. Since they use the glucose and fructose released from sucrose hydrolysis carried out by yeasts, their highest activity is expected to occur between days 3 and 10 of fermentation, when pH values have already decreased. In the case of the kombuchas developed in this study, they constitute the group showing the least fluctuation throughout the process, although it is worth highlighting the higher initial concentrations in the SC3-kombucha, which also shows the highest counts on day 7 (7.8 log CFU/mL).

The results obtained, although preliminary—as previously noted—encourage further investigation to deepen the taxonomic identification of the microorganisms present in kombucha and to elucidate their individual dynamics throughout the production process. Moreover, given that they directly influence not only the physicochemical, biochemical, and functional characteristics of kombucha but also its organoleptic properties, sensory analyses of the final products would be of great interest, particularly in relation to the predominance of specific microbial groups.

### 3.4. In Vitro Biological Activities of Selected Kombucha Beverages

Following the physicochemical, biochemical and microbiological characterization of the kombucha beverages, the most appropriate fermentation time point was selected for each formulation based on the parameters analyzed, corresponding to the suggested bottling stage. In this selection process, several criteria were considered: the pH should not be excessively acidic; a reduction in TSS content, and therefore in sugar content, should have occurred; and the kombuchas should exhibit maximum TPC values and, to a lesser extent, maximum soluble protein content. All these factors were evaluated while also considering the typical kombucha bottling period, which generally occurs between days 7 and 10 of fermentation. Accordingly, day 10 was selected for SC1- and SC2-kombuchas, whereas day 7 was selected for SC3- and SC4-kombuchas. These samples were subsequently subjected to in vitro antioxidant and antimicrobial activity analyses.

#### 3.4.1. Radical Scavenging Activity of the Selected Kombucha Beverages

As a preliminary approach to evaluate the antioxidant potential of the beverages obtained, in vitro antioxidant activity assays were performed, specifically assessing the DPPH^●^ and ABTS^●+^ scavenging capacities of the selected products. Although the results obtained are promising, they should be interpreted with caution and highlight the need for future studies employing cellular and in vivo models that more accurately reproduce real physiological contexts [37].

**Figure 6:**
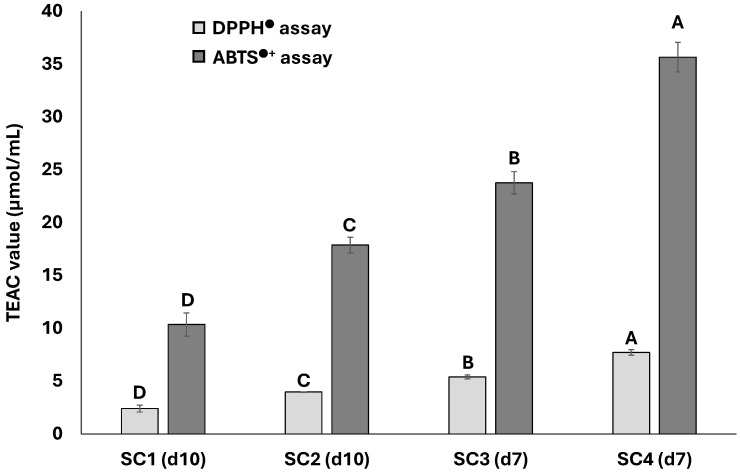
TEAC values (µmol/mL) of the selected kombuchas fermented with SC1, SC2, SC3 and SC4 at day 7 (d7) and day 10 (d10) obtained through DPPH^●^ and ABTS^●+^ assays. Different letters (A–D) denote significant differences between kombucha beverages for the same test (one-way ANOVA, Tukey’s test, *p* ≤ 0.05).

The kombuchas exhibited different radical scavenging capacities, although the same trend was observed in both assays (Figure 6). In decreasing order of TEAC values, the kombuchas ranked as follows: SC4, SC3, SC2, and SC1. Particularly noteworthy is the correlation between TEAC values and TPC of the kombuchas, given that, as previously mentioned, (poly)phenols are considered the main antioxidant compounds in tea kombucha beverages [38]. However, although moderately high R^2^ values were obtained between TPC and TEAC values (0.86 and 0.84 for DPPH^●^ and ABTS^●+^, respectively), the relationship was inversely proportional; that is, the beverages with lower TPC values exhibited significantly higher radical scavenging capacities. This unexpected result may be explained by several factors. First, the phenolic profile of kombucha should be considered, as a higher TPC does not necessarily imply the presence of specific (poly)phenols with strong antioxidant activity; therefore, future studies involving chromatographic analyses of the phenolic composition of these beverages are strongly recommended. Furthermore, (poly)phenols are not the only compounds present in tea kombucha with high antioxidant potential. Other molecules, such as ascorbic acid, DSL, and certain peptides of plant or microbial origin, may also contribute substantially to the antioxidant properties of kombucha beverages. Therefore, further identification and quantification analyses of these bioactive compounds are crucial to explain the higher antioxidant potential of SC4-kombucha [16,39].

Another remarkable aspect is the difference in TEAC values obtained between the two assays. Although the overall trend was highly similar, and a very strong correlation was observed between the TEAC values obtained for the different radicals (R^2^ = 0.999), the radical scavenging activities measured using the ABTS^●+^ assay were consistently higher, ranging from 4.3 to 4.6 times those obtained with DPPH^●^. This finding may be explained by the predominance of hydrophilic antioxidant molecules in kombucha beverages, which are able to interact more efficiently with ABTS^●+^ in an aqueous environment than with DPPH^●^ in a methanolic medium. Moreover, the underestimation of scavenging activity in the DPPH^●^ assay may result from an overestimation of the absorbance at 517 nm caused by the presence of green tea pigments [14].

Comparison of these results with those reported in the scientific literature confirms that the values obtained fall within the range previously described and published for other tea kombuchas. For instance, Cardoso et al. (2020) reported TEAC values of 8.2 and 13.6 µmol/mL in an ABTS^●+^ assay for green and black tea kombuchas, respectively, values comparable to those observed for SC1- and SC-2 kombuchas, but significantly lower than those obtained for SC3- and SC4-kombuchas (23.4 and 35.6 µmol/mL, respectively) [16]. Nevertheless, TEAC values vary considerably depending on the SC and fermentation conditions employed, ranging from 2 to 4 µmol/mL against DPPH^●^ in green tea kombuchas produced using a consortium similar to SC3 [7], to extremely high values approaching 7000 and 10,000 µmol/mL against DPPH^●^ and ABTS^●+^, respectively, in green and black tea kombuchas [40].

#### 3.4.2. Antimicrobial Activity of the Selected Kombucha Beverages

With the aim of conducting an initial screening, the potential antimicrobial activity of the selected kombuchas was evaluated against indicator strains, including non-pathogenic laboratory strains and reference strains from species associated with human pathogenicity. The disc diffusion method was employed, providing a preliminary qualitative approach that does not yield robust quantitative results. Therefore, the findings obtained should be validated in future studies using alternative methodologies capable of providing quantitative and more definitive conclusions.

However, the present study demonstrated that the kombuchas exhibited inhibitory activity against the tested strain of *Escherichia coli* (Table 2), with SC2-kombucha producing a significantly larger inhibition halo than the other beverages evaluated. Nevertheless, as previously mentioned, quantitative comparisons of the antimicrobial effects of kombuchas should be further investigated using more appropriate methodologies. In contrast, no inhibition halos were observed against the tested strains of *Staphylococcus aureus* and *Listeria innocua*. These findings differ from those reported in previous studies. For instance, Cardoso et al. (2020) observed inhibitory activity of green tea kombuchas against strains of *E. coli*, *S. aureus* and *Listeria monocytogenes*, reporting a minimum inhibitory concentration (MIC) of 250 µL/mL [16]. These discrepancies may be attributable to the limitations of the protocol employed in the present study. On the one hand, the concentration tested may have been below the MIC values required to inhibit the evaluated strains, highlighting the need for future studies aimed at determining the MIC of each kombucha against each bacterial strain. On the other hand, even within the same genus or species, bacterial susceptibility to the antimicrobial activity of kombuchas may vary depending on the specific strain used. Consequently, it would be of interest to expand the screening to additional strains of these species relevant to food safety [41]. Furthermore, fermentation conditions, the SC employed, and the phenolic composition of the kombuchas—particularly the concentration of specific (poly)phenols with antimicrobial activity—may positively or negatively influence the bioactivity of these beverages. Therefore, a comprehensive characterization of kombucha beverages is also recommended in order to better define their phenolic profiles and establish correlations between specific compounds and biological activities [42].

## 4. Conclusions

By applying the methodologies described, it was possible to analyze and monitor the physicochemical, biochemical, microbiological, and functional profiles of tea kombuchas fermented with different SCs over a 21-day period. The results suggested the significant impact of microbial consortium composition on beverage characteristics and enabled the selection of the most appropriate bottling stage depending on the SC employed.

The kombuchas obtained remained within the pH range generally considered safe (2.5–4.2) throughout the fermentation process, with the exception of SC3-kombucha, which exhibited a slightly higher initial pH value (4.27). No growth of undesirable microorganisms, such as moulds, was observed during fermentation. All kombuchas showed a reduction in TSS content associated with a decrease in soluble sugar concentration, reaching values between 3.2 and 4.6 °Brix during the bottling period (days 7–10). These results suggest that the beverages developed may represent a low-sugar alternative to other commonly consumed beverages, such as fruit juices and carbonated soft drinks. During the bottling period, increases in soluble protein concentration and TPC were also observed. Ethanol levels remained below the regulatory threshold of 1.2% in all kombuchas, except for SC2-kombucha, which reached exactly this value on day 7, supporting the recommendation to bottle this beverage on day 10 instead. Microbiological analyses provided a general overview of the dynamics of the main microbial populations throughout the different stages of fermentation, highlighting the increase in yeast counts during the early fermentation stages and the growth of AAB coinciding with periods of maximum ethanol availability in the beverages. Regarding the potential biological activities of the kombuchas, relevant TEAC values were obtained against both DPPH^●^ and, particularly, ABTS^●+^ radicals, with SC4-kombucha at day 7 showing the highest antioxidant activity. Furthermore, all four kombuchas exhibited inhibitory activity against the evaluated strain of *E. coli* at the selected bottling stage.

Overall, the results obtained can be considered highly promising, although they require further validation through additional studies. In this regard, the phenolic characterization of kombuchas should be expanded through the application of chromatographic techniques aimed at identifying and quantifying the specific (poly)phenols present in the beverages. Moreover, organic acids production and sugar consumption must be monitored through more sophisticated techniques. Likewise, future microbiological characterization should focus on the precise identification of the microbial genera and species present at each stage of fermentation. In terms of biological activities, antioxidant activity should be further evaluated using cellular and in vivo models that more accurately reproduce physiological conditions, while antimicrobial activity should be complemented with quantitative studies, such as MIC determination assays. If future studies validate the findings of this initial screening, sensory evaluation and consumer acceptance studies should also be encouraged in order to assess the feasibility of developing tea kombuchas fermented with these SCOBYs as potential functional beverages.

## Figures and Tables

**Table 1:** Evolution of ethanol content in kombuchas during fermentation (21 days) with SC1, SC2, SC3, and SC4. Different letters denote significant differences for the same SC at different fermentation times (A–D) and for different SCs at the same fermentation time (a,b) (one-way ANOVA, Tukey’s test, *p* ≤ 0.05).

	Ethanol Content (% *v*/*v*)					
	Day 0	Day 3	Day 7	Day 10	Day 14	Day 21
**SC1**	n.d. ^B,a^	0.30 ± 0.14 ^A,b^	0.40 ± 0.00 ^A,b^	0.20 ± 0.00 ^AB,b^	n.d. ^B,b^	n.d. ^B,a^
**SC2**	n.d. ^D,a^	1.65 ± 0.07 ^A,a^	1.20 ± 0.00 ^B,a^	0.70 ± 0.14 ^C,a^	n.d. ^D,b^	n.d. ^D,a^
**SC3**	n.d. ^D,a^	1.50 ± 0.14 ^A,a^	1.10 ± 0.14 ^B,a^	0.85 ± 0.07 ^BC,a^	0.70 ± 0.00 ^C,a^	n.d. ^D,a^
**SC4**	n.d. ^B,a^	n.d. ^B,b^	0.35 ± 0.07 ^A,b^	0.15 ± 0.07 ^BC,a^	n.d. ^B,b^	n.d. ^B,a^

**Table 2:** Inhibitory activity of selected kombucha elaborated with SC1, SC2, SC3 and SC4 at days 7 (d7) and 10 (d10) against *Escherichia coli* DH5α (EC), *Staphylococcus aureus* CECT86 (SA) and *Listeria innocua* SA1 (LI). Different letters (A–D) indicate statistical significance (*p* ≤ 0.05) between sizes of the inhibition zones (mm) of the same microorganism. “-” = No inhibition zone was observed.

	Size of the Inhibition Zones (mm)		
Sample	EC	SA	LI
**SC1 (d10)**	8.15 ± 0.21 ^C^	- ^B^	- ^B^
**SC2 (d10)**	9.65 ± 0.21 ^B^	- ^B^	- ^B^
**SC3 (d7)**	7.50 ± 0.00 ^C^	- ^B^	- ^B^
**SC4 (d7)**	6.65 ± 0.21 ^D^	- ^B^	- ^B^
**Gentamicin (10 µg)**	14.57 ± 0.12 ^A^	14.02 ± 0.06 ^A^	15.12 ± 0.88 ^A^

## Data Availability

The original contributions presented in the study are included in the article/Supplementary Materials; further inquiries can be directed to the corresponding authors.

## Supplementary Materials

The following supporting information can be downloaded at: https://www.mdpi.com/article/10.3390/foods15142445/s1, Figure S1: Microbial populations counts (log CFU/g) in the used SCs: Aerobic mesophilic microorganisms (AMM), yeasts, acetic acid bacteria (AAB), lactic acid bacteria (LAB). Different letters (A,B) denote significant differences between SCs for the same microbial group (one-way ANOVA, Tukey’s test, *p* ≤ 0.05).

## Abbreviations

The following abbreviations are used in this manuscript:

SCOBY/SC	Symbiotic Culture/Consortium of Bacteria and Yeasts
TSS	Total soluble solids
TPC	Total phenolic content
TEAC	Trolox equivalent antioxidant capacity
LAB	Lactic acid bacteria
AAB	Acetic acid bacteria
