# Supplementary material for: Microbial Consortia-Dependent Evolution of Physicochemical, Compositional and Functional Properties in Kombucha Fermentation
Source: Foods. 2026 Jul 9;15(14):2445. doi: 10.3390/foods15142445 (PMC13408931; doi:10.3390/foods15142445)
Supplement: Supplementary file 1 [file foods-15-02445-s001.zip › foods-4408710-supplementary.pdf]

Supplementary material

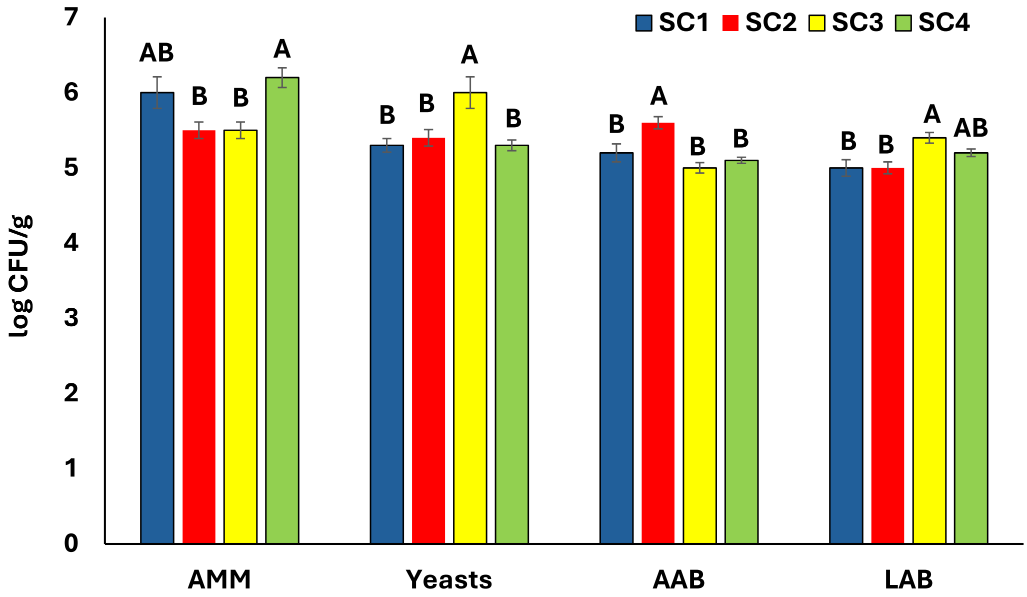

Supplementary Figure S1. Microbial populations counts (log CFU/g) in the used SCs: Aerobic mesophilic microorganisms (AMM), yeasts, acetic acid bacteria (AAB), lactic acid bacteria (LAB). Different letters (A-B) denote significant differences between SCs for the same microbial group (one-way ANOVA, Tukey's test, $p \leq 0.05$).
